# New insights into physician burnout and turnover intent: a validated measure of physician fortitude

**DOI:** 10.1186/s12913-024-11186-7

**Published:** 2024-06-18

**Authors:** Laurence Weinzimmer, Stephen Hippler

**Affiliations:** 1 Caterpillar Inc. Endowed Professor of Management, Bradley University, Peoria, IL USA; 2 Department of Internal Medicine, University of Illinois College of Medicine, Peoria, IL USA

**Keywords:** Physician fortitude, Physician burnout, Turnover, Turnover intent

## Abstract

**Background:**

Given the increasing prevalence of the physician burnout, this study provides new insights into the antecedents driving burnout and turnover intent. By introducing the concept of physician fortitude, we develop a valid and statistically-reliable measure that increases our understanding of these issues.

**Methods:**

A two-sample design was employed. Using a sample of 909 physicians, Advanced Practice Providers (APPs) and healthcare leaders, exploratory factor analysis was employed to create a 12-item fortitude scale. In the second study, using a sample of 212 of practicing physicians, APPs and healthcare leaders, bivariate and tetrachoric correlations, and ordinary least square regression modeling were able to establish reliability and validity.

**Results:**

The fortitude scale shows sufficient reliability. Moreover, we found significant support for convergent and criterion-related validity. Fortitude was significantly related to all three subdimensions of burnout, including emotional exhaustion (*r* = -.62, *p* < .01), depersonalization (*r* = -.70, *p* < .01) and personal accomplishment (*r* = .65, *p* < .01), and turnover intent (*r* = -.55, *p* < .01). Moreover, the fortitude measure explained more variance in all three subdimensions of burnout and turnover intent compared to common measures, including grit, hardiness, mental toughness and resilience (*p* < .01).

**Conclusions:**

Results from this study empirically demonstrate that fortitude is significantly related to burnout, and turnover intent. This new fortitude measure adds a new perspective to assist in the development of more effective interventions. Opportunities for future research are discussed.

## Background

For nearly thirty years, researchers have sought to understand the antecedents and consequences of physician burnout, yet the problem continues. Notably, Shanafelt et al., found that 62.8% of physicians had at least one manifestation of burnout in 2021, compared with 45.5% in 2011 [1]. Consequently, patients have a higher likelihood of being treated by a burned-out physician today than one who is not [1, 2]. This is associated with decreased quality of care, medical errors, poor patient satisfaction, and limited patient access [3, 4]. Moreover, beyond the human cost of burnout on individuals, the financial cost of burnout related turnover on the healthcare system is estimated to be $2.6 to $6.3 billion (USD) a year, or $7,600 (USD) per employed physician [5]. Clearly more research needs to be conducted to understand the antecedents of physician burnout and turnover so that more effective strategies can be developed to address this growing problem.

### Overview of physician burnout

Existing research on physician burnout has primarily examined two sets of drivers, intrinsic personality traits and extrinsic work environments. Intrinsic personality traits such as high neuroticism, low agreeableness and low conscientiousness as measured by the Big 5 Inventory have been associated with an increased risk of burnout [6]. Likewise, extrinsic work environmental factors such as excessive workload, poor work-life balance, low autonomy and systemic barriers all contribute to burnout and intent to leave. [7, 8].

Much recent research focused on the importance of the work environment has led to interventions to improve physician wellbeing [9–12]. However, in a recent meta-analysis of 38 randomized trials using different interventions focused on improving physician burnout, results suggested these efforts did not result in meaningful impacts on clinical burnout [13]. Furthermore, the authors suggest that a more nuanced understanding of the causes of burnout is needed to develop more effective interventions. Similarly, Cataputo et al., looked at interventions focused on mitigating work-related stress in healthcare using cognitive behavioral therapy, relaxation therapy; and interventions focused on the organization. They concluded that individual-level interventions were beneficial over the short term, but organizational-level intervention failed to show any benefit in reducing burnout [14]. Although these studies have provided important insights into physician burnout, there are still significant opportunities to extend these findings.

Consequently, a critical question to consider is while it is estimated that up to 50% of physicians are suffering from burnout at any one time, why do 50% of physicians in presumably similar circumstances not burnout? One possible explanation may be that individuals can perceive their work environment very differently. In a study of UK house officers, differences in self-reported trainee stress levels were shown to be related to individual differences within the doctors themselves and not organizational factors present or the administrative structure of hospitals [15]. Furthermore, in a separate study, researchers determined how doctors perceived their workplace climate and workload can be partially predicted by trait measures of personality taken five years earlier [16]. That is not to say that factors such as work environment and systemic factors are unimportant but suggests that there is a need to build upon this past empirical research to increase our collective understanding of how an individual’s perspective and attitude may contribute to their unique responses to environmental stressors in healthcare.

Extant literature has yielded a significant amount of research assessing the relationship between intra-personal attributes with burnout and/or turnover across multiple professions. Specifically, there is a growing body of research that has demonstrated how grit [17–19], self-efficacy [20], hardiness [21–23], resilience [24, 25], mental toughness [26, 27] and hope [28, 29] individually mitigate the relationship between environmental stressors and burnout-related phenomena in healthcare and many other professions. While these unique individual attributes have proven to be beneficial, we believe there is a significant opportunity to integrate these constructs to develop a more holistic understanding of how individuals use all of these attributes to respond to their unique work-related stressors.

### Integration of concepts

There have been previous attempts to integrate the abovementioned attributes. The limited literature on *fortitude* shows promise as a unifying construct [30–32]. However, this body of research has been studied using different definitions, antecedents and outcome measures. For example, Pretorius et al. defined fortitude as an attitude to manage stress and stay well. He postulated that this strength derives from an appraisal of the self, the family and support from others. Their 20 item Fortitude Questionnaire (FORQ) was tested and validated in undergraduate psychology students [31]. Henttonen et al. attempted to measure fortitude by developing a scale based on the Finnish cultural attribute of *sisu*, defined as determination and resoluteness in the face of adversity. They combined multiple intrapersonal attributes with personality traits to develop a 18-item scale in a general working population [30]. Similarly, VanTongeren et al. developed a validated measure for spiritual fortitude. Their measure includes items for spiritual enterprise, spiritual endurance and redemptive purpose and was validated in a volunteer population [32].

While there has been significant value from the fortitude research, its applicability and generalizability to physician burnout and turnover is limited. To advance the literature and extend our understanding of physician burnout, there is a need to arrive at a more precise definition relevant to healthcare. Subsequently, we suggest fortitude to be an interpersonal attitudinal attribute that enables one to succeed under repeated pressure and stress. Therefore, the purpose of this study is to gain new insights into burnout and turnover by investigating fortitude in healthcare and developing a statistically valid and reliable scale.

## Methods

To assess the potential impact of physician fortitude on burnout and turnover intent, we drew on previous research that has focused on explaining how individuals overcome adversity. We employed a two-sample design to establish content validity, internal consistency, empirical reliability, unidimensionality, convergent validity and ultimately criterion-related validity. Surveys were approved by the University of Illinois College of Medicine Institutional Review Board. Informed consent was attained by all survey participants prior to completion of the survey.

### Item generation

To ensure we met the psychometric assumptions proposed by our latent construct we define as fortitude, we used a *deductive* approach [33]. Grounded in extant literatures that have shown empirical promise regarding how individuals experience and ultimately overcome adversity, seven specific areas of research were identified. Specifically, we drew on grit, hardiness, mental toughness, resilience, hope, optimism and self-efficacy to generate a potential list of items for our integrative fortitude construct. Based on commonalities across these dimensions, 64 potential items were identified. We then engaged 73 physicians and non-physician healthcare leaders in 13 focus groups to provide feedback on the suitability of the potential items in a healthcare context. Based on relevancy, these focus groups reduced the potential list of survey items to 45.

Next, we ensured sufficient interrater reliability to evaluate internal consistency of potential items for the fortitude scale [34]. A panel of five academic researchers actively engaged in burnout and turnover research were queried and asked to match potential survey items with the fortitude construct. Crocker and Algina define a minimum value greater than 0.70 to be acceptable for consistency estimates of interrater reliability [35]. Consequently, if an item had an interrater reliability value that did not meet the 0.70 threshold, it was deleted as a possible item for the fortitude scale. This resulted in an interrater reliability 0.88. Results from the interrater reliability assessment yielded 34 potential items.

### Data reduction, reliability and unidimensionality

To further reduce potential items, in our first study (study 1) we collected data from a sample of 909 practicing physicians, APPs and healthcare leaders from a large U.S. healthcare system. Respondents were asked to rate the degree to which each statement accurately described their own individual attributes on the 34 items using a seven-point Likert Scale, where 1 = strongly disagree and 7 = strongly agree. A seven-point Likert Scale was used, as this is most consistent with the literatures we used to develop our potential list of items.

Results from study 1 provided initial evidence of reliability, construct validity and unidimensionality. Exploratory factor analysis (EFA) was used to assess potential items. Given the data were normally distributed, a maximum likelihood extraction method and Varimax rotation were used [36]. We were able to identify potential items that had individual factor loadings greater than 0.70. This yielded a 12-item scale measuring fortitude in healthcare (HCF-12). Example items include “I am excited about working on achieving my goals,” “I am determined to succeed in achieving my goals,” “I am passionate about the work I do.”

The EFA provided initial support for the unidimensionality of a single factor for the HCF-12 scale, as well as initial construct validity. Specifically, scree plots indicated unidimensionality for our HCF-12 measure. The Kaiser–Meyer–Olkin (KMO) measure of sampling adequacy was 0.88, well above the suggested threshold of 0.5 [37], and Bartlett’s test of sphericity was significant (*p* < 0.05). Initial reliability was encouraging, yielding a Cronbach alpha of 0.89.

### Convergent and criterion-related validity

After finding encouraging evidence of reliability and unidimensionality, we collected data from a second sample (study 2) to measure convergent validity and criterion-related validity. The survey in study 2 was sent to practicing physicians and healthcare leaders (*n* = 212). Similar to Study 1, we found encouraging reliability with a Cronbach’s alpha = 0.93. Measures for internal consistency and factor loadings of the 12 items in the HCF-12 for Study 2 can be seen in Table 1.

**Table 1:** Internal consistency and factor loadings for HCF-12 – study 2

Item	Inter-Item Correlation	Cronbach’s Alpha if Item Deleted	Factor Loading
HCF1	.662	.928	.771
HCF2	.726	.924	.847
HCF3	.675	.925	.830
HCF4	.635	.925	.825
HCF5	.669	.928	.768
HCF6	.581	.929	.730
HCF7	.680	.927	.770
HCF8	.605	.929	.742
HCF9	.700	.930	.717
HCF10	.674	.930	.702
HCF11	.761	.930	.712
HCF12	.676	.929	.736

*n* = 212

To test for convergent validity, Study 2 included the HCF-12 scale and scales grit [17], hardiness [21], resilience [24], mental toughness [27]. To establish criterion-related validity, the survey also included scales for all three subdimensions of burnout (emotional exhaustion, depersonalization, and personal achievement [38], and turnover intent [39], as previous literature has shown that chronic burnout leads to turnover [40–42]. Additionally, based on focus-group insights, control variables were added including age, gender, race, hours worked and call burden. Note that reliabilities for all scales used in Study 2 are included in Table 2.

**Table 2:** Correlations, means, std. dev and reliabilities – study 2

Variable	Mean	SD	1	2	3	4	5	6	7	8	9	10
1. Turnover intent	2.78	1.71	(.90)									
2. Emotional Exhaustion	3.98	1.48	.57**	(.89)								
3. Depersonalization	3.24	1.66	.69**	.70**	(.89)							
4. Personal Accomplishment	5.99	0.79	-.50**	-.40**	-.54**	(.76)						
5. Fortitude	5.95	0.77	-.55**	-.62**	-.70**	.65**	(.93)					
6. Grit	5.31	0.75	-.04	.07	-.02	.15	.02	(.82)				
7. Hardiness	4.71	0.61	-.04	-.06	-.13	.21**	.28**	.44**	(.57)			
8. Mental Toughness	5.28	0.74	-.02	.23**	.10	.09	.03	.32**	.15	(.80)		
9. Resilience	6.04	0.61	-.28**	-.38**	-.40**	.58**	.71**	.01	.01	.12	(.89)	
10. Age	44.74	11.30	-.22**	-.27**	-.21**	.09	.24**	-.20*	-.18*	-.11	.07	(na)
11. Reported work hours	42.30	9.86	-.06	-.03	-.08	-.01	.04	.07	.01	.00	-.06	.54*

Personal accomplishment is reverse scaled

Reliability estimates (Cronbach’s alpha) appear on cross diagonal in parentheses

*n* = 212

^*^ *p* < 0.05

^**^ *p* < 0.01

Given the HCF-12 draws on attitudinal measures specifically related to stressors, including grit, hardiness, resilience and mental toughness, we would expect strong relationships among these variables. To assess convergent validity, we used correlational analyses. We found significant correlations between fortitude and hardiness (*r* = 0.28, p < 0.01) and resilience (*r* = 0.71, *p* < 0.01). However, there were not significant relationships between our fortitude measure with grit or mental toughness**.** To assess criterion-related validity, we would expect that the HCF-12 measure to be negatively related to burnout and turnover intent. Fortitude was significantly related all three subdimensions of burnout, specifically emotional exhaustion (*r* = -0.62, *p* < 0.01), depersonalization (*r* = -0.70, *p* < 0.01) and personal accomplishment (*r* = 0.65, *p* < 0.01), and turnover intent (*r* = -0.55, *p* < 0.01). Given that fortitude was significantly related to all of these constructs in the predicted directions, this provides strong initial evidence for criterion-related validity.

## Findings and results

Based on encouraging results regarding reliability and validities of the HCF-12, this creates a platform that allows us to compare fortitude with the scales currently recognized as antecedents in the extant burnout and turnover-intent literatures. Subsequently, we used ordinary least-squared (OLS) regression modeling to test significance levels and explained variance to compared the HCF-12 to grit, hardiness, mental toughness and resilience based on data from the 212 respondents in study 2,

### Sample characteristics

Of the 212 respondents who completed the survey, 76% were female, 21% were male and 3% preferred not to answer. For race, 91% of respondents were White, 5% were Asian, 2% were Hispanic and 2% were Black. The average age of respondents was 47.4 years old; they worked an average of 51.3 h per week and worked for an average of 8.8 years in the current hospital system. Finally, in terms of their role, respondents were asked if they were a physician leader (26%), a non-physician leader (49%) or a practicing clinician (29%).

### Descriptive statistics

Means, standard deviations, reliability scores, and bivariate correlations can be seen in Table 2. Note that reliabilities for all scales met the minimum threshold of 0.70 (with the exceptions of hardiness) and the HCF-12 had the highest reliability score of 0.93. Also note that the fortitude measure was significantly related to all three subdimensions of burnout and turnover intent, providing encouraging support for the consideration of the new scale.

### Ordinary least squares regression results

In order to compare the HCF-12 with existing measures, regression analyses were performed. Specifically, in Tables 3–6, fortitude, grit, hardiness, mental toughness and resilience were all regressed on turnover intent and the three subdimensions of burnout, namely emotional exhaustion, depersonalization and personal accomplishment.

**Table 3:** OLS simple regression analyses comparisons – Study 2

**Turnover Intent**					
**Interpersonal Attributes**					
Fortitude	-.55**				
Grit		-.23*			
Hardiness			-.31**		
Mental Toughness				-.27**	
Resilience					-.28**
R-squared	.31	.05	.10	.07	.08
Adj. R-squared	.30	.04	.09	.06	.07
F	67.44**	8.22*	16.65**	12.67**	13.93**

Standardized Beta Coefficients are provided

*n* = 212

^*^ *p* < 0.05

^**^ *p* < 0.01

In Table 3, all five scales were negatively related with turnover intent. Specifically, fortitude (β = -0.55), grit (β = -0.23) hardiness (β = -0.31), mental toughness (β = -0.27) and resilience (β = -0.28) were all statistically significant (*p* < 0.01). Note that there was a statistically significant difference in explained variance between fortitude and all of the other measures (*p* < 0.01). The adjusted R^2^ for fortitude was 0.30 compared to grit (0.04), hardiness (0.09), mental toughness (0.06) and resilience (0.07).

In Table 4, all five scales were negatively related with the burnout subdimension of emotional exhaustion. Specifically, fortitude (β = -0.62), grit (β = -0.35) hardiness (β = -0.56), mental toughness (β = -0.45) and resilience (β = -0.38) were all statistically significant (*p* < 0.01). Note that there was a statistically significant difference in explained variance between fortitude and all of the other measures (*p* < 0.01). The adjusted R^2^ for fortitude was 0.39 compared to grit (0.12), hardiness (0.32), mental toughness (0.29) and resilience (0.15).

**Table 4:** OLS simple regression analyses comparisons – Study 2

**Emotional Exhaustion**					
**Interpersonal Attributes**					
Fortitude	-.62**				
Grit		-.35**			
Hardiness			-.56**		
Mental Toughness				-.45**	
Resilience					-.38 **
R-squared	.39	.12	.32	.29	.15
Adj. R-squared	.38	.11	.31	.28	.14
F	94.28**	20.63**	72.16**	63.59**	26.35**

Standardized Beta Coefficients are provided

*n* = 212

^*^ *p* < 0.05

^**^ *p* < 0.01

In Table 5, all five scales were negatively related with the burnout subdimension of depersonalization. Specifically, fortitude (β = -0.69), grit (β = -0.36) hardiness (β = -0.50), mental toughness (β = -0.47) and resilience (β = -0.40) were all statistically significant (*p* < 0.01). Note that there was a statistically significant difference in explained variance between fortitude and all of the other measures (*p* < 0.01). The adjusted R^2^ for fortitude was 0.48 compared to grit (0.13), hardiness (0.25), mental toughness (0.22) and resilience (0.16).

**Table 5:** OLS simple regression analyses comparisons – Study 2

**Depersonalization**					
**Interpersonal Attributes**					
Fortitude	-.69**				
Grit		-.36**			
Hardiness			-.50**		
Mental Toughness				-.47**	
Resilience					-.40 **
R-squared	.48	.13	.25	.22	.16
Adj. R-squared	.47	.12	.24	.21	.15
F	138.21**	22.27**	52.55**	44.84**	29.77**

Note: Standardized Beta Coefficients are provided

*n* = 212

^*^ *p* < 0.05

^**^ *p* < 0.01

In Table 6, all five scales were positively related with the burnout subdimension of personal accomplishment. Specifically, fortitude (β = 0.65), grit (β = 0.41) hardiness (β = 0.49), mental toughness (β = 0.49) and resilience (β = 0.59) were all statistically significant (*p* < 0.01). Note that there was a statistically significant difference in explained variance between fortitude and all of the other measures (*p* < 0.01). The adjusted R^2^ for fortitude was 0.42 compared to grit (0.16), hardiness (0.23), mental toughness (0.23) and resilience (0.34).

**Table 6:** OLS simple regression analyses comparisons – study 2

**Personal Accomplishment**					
**Interpersonal Attributes**					
Fortitude	.65**				
Grit		.41**			
Hardiness			.49**		
Mental Toughness				.49**	
Resilience					.59**
R-squared	.42	.17	.24	.24	.35
Adj. R-squared	.42	.16	.23	.23	.34
F	111.89**	29.96**	49.38**	48.81**	83.66**

Standardized Beta Coefficients are provided

*n* = 212

^*^ *p* < 0.05

^**^ *p* < 0.01

In summary, fortitude explained significantly more variance in turnover intent, emotional exhaustion, depersonalization and personal accomplishment than any of the existing measures.

## Discussion

The primary focus of this research was to investigate the potential use of a new fortitude scale to increase our understanding of antecedents to physician burnout and turnover intent. Results from this study provide encouraging evidence that the integration of grit, hardiness, mental toughness and resilience, in a latent the construct defined as fortitude, provides new insights into understanding how the combination of these intrapersonal attributes contribute to physician burnout and turnover intent. Our proposed HCF-12 scale exhibited encouraging findings to support the psychometric properties of a physician fortitude scale. Specifically, based on a two-study design, we were able to establish content validity, unidimensionality, empirical reliability, convergent validity and criterion-related validity. Moreover, we were able to show that the HCF-12 resulted in significantly higher levels of explained variances for all three subdimensions of burnout and turnover intent when compared to all existing scales found in the extant literature. Consequently, findings from our study provide additional insights into understanding physician burnout and turnover intent. Specifically, our findings make several contributions.

First, our findings extend previous fortitude research that showed the combination of different psychological antecedents of well-being provide additional insights beyond single constructs alone. Specifically, Pretorius et al. derived their measure from the constructs of hardiness, sense of cohesion and potency and found that fortitude, defined as the strength to manage stress and to stay well, was significantly related to students’ well-being and distress [31]. Similarly, Henttonen et al. combined attributes of mental toughness, grit, hardiness, resilience, hope and self-efficacy with personality traits and studied the effects of beneficial and harmful *sisu* on the happiness and wellbeing of survey participants [30]. Likewise, VanTongeren et. al. developed a unique scale for spiritual fortitude which predicted variance in meaning in life, spiritual well-being, religious coping and adversity-related anxiety [32]. Our study and the Healthcare Fortitude Scale (HF-12) extends these studies conceptually and relies on well-established constructs and validated instruments. Furthermore, we validate this scale so that it is applicable to healthcare workers.

Second, our findings support and extend research examining the impact of resilience on burnout [24, 25]. In a study by Roslen et. al. the authors contend the concept of resilience has unfortunately been used interchangeably with mental toughness, hardiness, and grit, adding to confusion in the literature [43]. Stoffel and Cain likewise state that it is often difficult to tell whether the constructs of resilience, grit, hardiness and mental toughness are distinct from each other as some authors use these terms interchangeably [44]. Consequently, by combining these concepts using the HCF-12, our findings support and extend this research by empirically demonstrating that an elevated concept of fortitude is a better and more precise construct than an expanded definition of resilience. Furthermore, our fortitude measure explains more variance than any of the individual concepts, including resilience alone, proving that the combination of constructs is better than any single one alone.

Third, the findings from this study may help to change the conversation regarding physician burnout and turnover intent. Our work suggests that the interpersonal attribute of fortitude leads to decreased burnout and turnover intent. Moreover, fortitude can be viewed as attitudinal and therefore malleable when compared to personality traits. Subsequently, it may help explain the interaction between each individual and their work environment. As such, interventions that are mindful of empowering individuals to develop fortitude in addition to changes in the work environment may lead to faster progress in mitigating this important issue in healthcare.

## Limitations and future research

We recognize potential limitations of the current study. First, we used a cross-sectional sample, collected at one point in time. Second, we recognize the possibility of common-methods bias, even though cross-sectional sampling is considered an acceptable method of collecting perceptual data [45].

Future research should strive to obtain longitudinal data and sources of secondary data to improve criterion-related validity. Likewise, future research on fortitude may want to consider the impact of fortitude on organizational context measures, such as perceived supervisor support (PSS) and organizational culture. It may not be the environment or the person alone, but the interaction between the adequacy of the multiple skills and attributes of the individual with ever evolving demands of the environment that contributes to wellbeing. Given the encouraging measures of validity and reliability of the HCF-12, future research can also test the moderating and mediating roles of fortitude on the relationship between work stress and burnout, work stress and turnover intent and burnout and turnover intent. Finally, this research was performed on a U.S.-based sample. Future research can assess fortitude among physicians in other countries to increase generalizability.

## Conclusion

This study represents the first attempt to define and measure fortitude using a U.S.-based sample in a healthcare environment. Moreover, this study develops a valid, reliable and generalizable scale that extends our understanding of antecedents that lead to physician burnout and turnover intent. As a malleable attribute, fortitude also provides new opportunities for targeted intervention strategies to improve physician wellbeing.

## Data Availability

No datasets were generated or analysed during the current study.
